# Report of Discussion on Ohio State Dental Society

**Published:** 1890-12

**Authors:** 


### Ohio State Dental Society.—Report of Discussion on

DR. F. W. SAGE'S PAPER.—TUESDAY AFTERNOON, OCT. 28TH, 1890.

After the reading of Dr. Sage's paper, Dr. Otto Arnold said: "I am very much delighted with this paper. It presents many valuable practical suggestions. We have all been puzzled by the discovery of just such complications—when attempting a diagnosis of difficult cases—as are therein described. As regards the principles of diagnosis much remains to be learned. The science is still in its infancy. We have no infallible rules laid down, by following which we may be sure of arriving at a position to grasp all the details of any disease; to recognize and identify it in all its varied phases. I myself have had a number of cases in which the symptoms were difficult of recognition and the treatment consequently not clearly indicated. I recall the case of a lady confined to her bed by what her physician supposed was diphtheria. After being under treatment for a week she began to suspect that her teeth were in some way implicated. I was called in and made an examination; found the pharynx and tonsils so severely inflamed that it was with some difficulty that I at last decided that the prime source of her trouble was in an impacted wisdom tooth. Upon extracting that she gradually but surely improved and soon got well. In another case of an external opening of an abscess in the lower jaw—the fistula being near the mental foramen—a physician had extracted the two lower 1st and 2nd molars without affording any relief. The wisdom tooth only remained on that side. This gave no response to the usual test for a dead pulp, nor could the fistula be certainly traced back to it. This tooth being of little value on account of the loss of its antagonist, was removed, experimentally, and most happily as it proved, for the fistula soon healed.

Dr. M. H. Fletcher: There are some very practical points in this paper. Ability to correctly diagnosticate would be, to my mind, perhaps a crucial test—if such a test were required—of our claim as professional men to rank as compeers of men in other professions. Such cases as are cited in the paper, and by Dr. Arnold are difficult to diagnosticate. Cases arise that tax to the utmost our facilities of observation and insight, our judgment and our ability to cope with the complications. Dr. Sage will recall a case that I now refer to, in which after three or four operations for the removal of necrosed bone pertaining to the superior maxilla, pain which had at first and for a long time appeared to be the result of the necrosis, at length located itself in the teeth, to the mystifying of the surgeon in charge. I found upon after examination, what I took to be something peculiar in the appearance of the pulps in one or two teeth that had been extracted. Upon examination of sections with the microscope I discovered unmistakable evidence of infiltration of the coloring matter of the blood into the connective tissue of the pulp forming zones or congested tracts. The pains have hitherto been confined to the upper jaw, but of late the patient complaines of trouble commencing in sound teeth of the lower jaw. I sometimes suspect, however, that these expressions of pain are largely imaginary on the part of this particular patient.

Dr. Barnes: I had a lady patient twenty-five years of age who gave birth to a seven month's child. Puerperal fever set in and her life was despaired of. Several of the best physicians in Cleveland attended her. The patient's face was swollen on both sides. The physicians suspected blood-poisoning, but I pointed out that she was erupting wisdom teeth. She had no return of the glandular swelling on the occasion of the birth of a second child later. Another case: A lady suffered for eighteen years with a fistulous opening between a bicuspid and a molar. Her own brother, a graduate of a New York dental school, operated for her relief, but unsuccessfully. She came to me. I found the second molar wanting, and she volunteered the information that her physician had extracted it (calling it by name) several years before. I found, however, this second molar encysted, and extracted it, relieving the discharge, shortly. It appeared upon searching inquiry that her physician had extracted a temporary molar. In another case recently brought to my notice the patient, a gentleman, had been treated eighteen months for a fistula discharging upon his shoulder. Blood disease of some sort was suspected. I extracted a lower molar that gave evidence of being implicated, and he soon showed very marked signs of improvement.

Dr. C. R. Butler: A robust-looking man, past middle life, was threatened with what his physicians called carbuncles. The patient himself had suspected his teeth had something to do with his pains, but upon consulting two or three dentists had been told that he was growing old, and that it was not worth while trying to do any thing with his teeth. Finally a physician sent him to me. I found the pulps dead in three teeth. By treating these he was relieved of threatening symptoms of septicæmia lately noticed. Lest it should appear that septicæmia would hardly arise from such a source, I wish to emphasize the fact that this disorder does frequently result from a very small amount of effete matter.

[SUBJECT PASSED].

